# New Insights into Chemoresistance Mediated by Mdm2 Inhibitors: The Benefits of Targeted Therapy over Common Cytostatics

**DOI:** 10.3390/biomedicines12030547

**Published:** 2024-02-29

**Authors:** Tatyana Grigoreva, Aleksandra Sagaidak, Daria Novikova, Vyacheslav Tribulovich

**Affiliations:** Laboratory of Molecular Pharmacology, St. Petersburg State Institute of Technology (Technical University), St. Petersburg 190013, Russia; aleksandrasagaidak@yandex.ru (A.S.); dc.novikova@gmail.com (D.N.)

**Keywords:** Nutlin-3a, paclitaxel, cytostatics, drug resistance, MDR, P-glycoprotein, BCRP

## Abstract

The inhibition of the Mdm2-p53 protein–protein interaction is a promising strategy for anticancer therapy. However, the problem of developing secondary chemoresistance in tumors treated with such drugs has not yet been sufficiently studied. In this work, we compared the properties of a drug-resistant cell line obtained during long-term cultivation in the presence of an Mdm2 inhibitor, Nutlin-3a, with a similarly obtained line insensitive to the cytostatic drug paclitaxel. We first confirmed the higher safety levels of Mdm2 inhibitors when compared with cytostatics in terms of the development of secondary chemoresistance. We showed that Nutlin-3a affects both the targeted p53-mediated cellular machinery and the universal ABC-mediated efflux mechanism. While both targeted and general defense mechanisms are activated by the Mdm2 inhibitor, it still increases the susceptibility of tumor cells to other drugs. The results obtained indicate that the risks of developing chemoresistance under the therapy with a targeted agent are fundamentally lower than during cytotoxic therapy.

## 1. Introduction

Targeting the physical protein–protein interaction between the p53 tumor suppressor protein and its E3 ubiquitin-protein ligase Mdm2 (EC 2.3.2.27) represents an attractive strategy for the treatment of cancers. Although monotherapy with such drugs has not shown positive results yet, their combined therapy with chemotherapeutic drugs or other targeted agents seems promising, which has been reflected in numerous clinical trials [1]. Although such targeted drugs are expected to be safer than chemotherapy [2], there is still a probability of primary and secondary resistance, which ultimately leads to treatment failure, as in the case of colorectal cancer, one of the most common and lethal types of tumors in both men and women worldwide [3]. The fact of resistance development was noted for a number of Mdm2 inhibitors, including RG7388 [4], MI-63 [5], HDM201 [6], idasanutlin [7], and SAR405838 (MI-77301) [8].

The development of resistance may be related to specific mechanisms targeted by a drug. In particular, resistance to Mdm2 inhibitors is usually associated with mutations in the p53 protein; the drug can both select for p53-mutated cells (Nutlin-3a, [9]) and induce de novo p53 mutations (Nutlin-3 [10], SAR405838 [8], MI-63 [11]). All Mdm2-p53 PPI inhibitors share a single pharmacophore hypothesis, which is based on mimicking the three key amino acids of the p53 alpha helix bound by the Mdm2 cavity [12]. Consequently, the resistance developed in response to such a drug should extend to all Mdm2 inhibitors.

Indeed, as shown in [11], MI-63 and Nutlin-3a provoke similar cross-resistance, but also cause a slight decrease in the sensitivity to doxorubicin and bortezomib, which act through other mechanisms. The phenomenon of such multidrug resistance (MDR), whereby acquired resistance to one drug reduces the susceptibility to other drugs that act on the tumor through other mechanisms, represents a huge problem in chemotherapy [13,14]. However, this is practically not taken into account when developing targeted Mdm2 inhibitors; the interest of researchers in this area is entirely focused on the changes in p53 itself.

In this work, we focused on assessing the risks of developing the multidrug resistance of tumor cells when treated with Nutlin-3a. Since understanding the mechanisms of acquired drug resistance to targeted therapies is critical for the development of novel, rational, and more effective treatment combinations [3], in this paper, we studied in detail the properties of a chemo-resistant line obtained as a result of selection using Nutlin-3a, comparing it with a line established in the presence of the classical cytotoxic drug paclitaxel. While Nutlin-3a triggers cell apoptosis by reactivating the pro-apoptotic protein p53 [15,16], paclitaxel stabilizes microtubules by disrupting spindle formation, which leads to cell cycle arrest in the G2/M phase, as well as cell death [17,18]. Both lines were obtained via monthslong cultivation with a gradual increase in the concentration of the corresponding drug in the medium, and they fully adapted to them, as evidenced by the growth rate corresponding to the original HCT116 line.

## 2. Materials and Methods

### 2.1. Reagents, Primers and Antibodies

Paclitaxel (taxol), a cytostatic anticancer drug, Nutlin-3a, an inhibitor of the Mdm2 protein, tariquidar and sodium orthovanadate (Na_3_VO_4_), inhibitors of ABC transporters, and DMSO were purchased from Sigma-Aldrich (St. Louis, MO, USA); Hoechst 33342 was obtained from Invitrogen Corporation (Waltham, MA, USA); Rhodamine 123 and MTT reagent ((3-(4,5-dimethylthiazole-2-yl)-2,5-diphenyl tetrazolium bromide) were purchased from MedChem Express (Monmouth Junction, NJ, USA). DMEM, Opti-MEM, *L*-glutamine, and FBS were purchased from Gibco, Thermo Fisher (Waltham, MA, USA).

The stock solutions of paclitaxel, Nutlin-3a, tariquidar sodium orthovanadate, Rhodamine 123, and Hoechst 33342 were prepared in DMSO.

Antibodies from Santa Cruz Biotechnology (Dallas, TX, USA) and Abcam (Cambridge, UK) were used. Primers were purchased from Evrogen (Moscow, Russia).

### 2.2. Cell Lines

The human colorectal carcinoma cell line (HCT116_wt_) was provided by the BIOCAD biotechnology company. This line is sensitive to both paclitaxel [19] and Mdm2 inhibitors [20]. Paclitaxel- and nutlin-resistant (HCT116_tax_ and HCT116_nut_, respectively) cell lines were established as described [21], using the described approaches [22,23].

Resistant and parental human colorectal carcinoma cell lines were stored under liquid nitrogen supplemented with 10% DMSO, and cultivated in DMEM supplemented with 10% FBS, 150 mg of dry *L*-glutamine at 37 °C, and 5% CO_2_, until a confluence of ≥80% was reached, followed by biological testing or subculturing using 0.25% trypsin. Cell lines were used at a low passage (<12 passages) for <4 months.

### 2.3. MTT Test, EC_50_

Cells were seeded into the wells of a 96-well plate at a density of ~100,000 cells per well in 100 µL of DMEM supplemented with *L*-glutamine and 10% FBS. After 24 h, the medium was exchanged in all wells by Opti-Mem supplemented with 4% FBS containing test substances or 0.5% DMSO as a control. Wells with the medium without cells were used as a blank. The plate was incubated for 48 h at 37 °C and 5% CO_2_, then the medium was aspirated, and 100 µL of 0.5 mg/mL MTT reagent solution was added to each well. After 4 h at 37 °C and 5% CO_2_, the medium was removed and formazan crystals were dissolved in 100 µL DMSO by mixing for 20 min. Absorption was measured at 560 nm in a microplate reader (CLARIOstar^®^, BMG LABTECH, Inc., Cary, NC, USA). The reference absorbance at 690 nm was used to correct for nonspecific background values.

Viability as a percentage was calculated according to the following Formula (1) [24]:

(1) $$Viability=\frac{{OD}_{treated}-{OD}_{blank}}{{OD}_{untreated}-{OD}_{blank}}\cdot 100\%,$$

where OD is the mean optical density at 560 nm.

### 2.4. Transport Activity

The assay was conducted as described earlier [15,21]. The cells were plated into a 96-well plate with a final density of ~50,000 cells/well, and cultured in DMEM supplemented with *L*-glutamine and 10% FBS for 24 h. Transport inhibitors, tariquidar [25] and Na_3_VO_4_ [26] (final concentrations 0.5 μM and 500 μM, respectively), were added to the wells in the Opti-Mem medium. Then, Hoechst 33342 (*C* = 1.25 μM) or Rhodamine 123 (*C* = 1 μM) was introduced into the wells for 30 min. Cells stained with the dyes in the absence of the inhibitor were used as controls. The cells were plated in triplicate. The plate was kept in the dark at room temperature on a shaker. After incubation with the drugs, the wells were washed with PBS, after which the Opti-Mem medium was added. The fluorescence measurements were carried out using a CLARIOstar^®^ microplate reader (BMG LABTECH, Inc., Cary, NC, USA). The Operetta CLS™ high-content analysis system (Perkin Elmer, Waltham, MA, USA) [27] was used for inoculation quality control (filters: brightfield (excitation transmission/emission 650–760 nm), Hoechst 33342 (excitation 360–400 nm/emission 410–480 nm)).

### 2.5. qPCR Analysis

The extraction of total RNA from cells was performed using the TRIzol reagent (Invitrogen Corporation, Waltham, MA, USA). cDNA synthesis was carried out using the MMLV RT kit (Evrogen, Moscow, Russia) according to the following protocol: 1 μg RNA was added to the reaction mixture (20 μL).

Real-time PCR was performed on a CFX96 Touch Real-Time RCR Detection System (Bio-Rad, United States) using the qPCRmix-HS SYBR kit (Evrogen, Moscow, Russia). The PCR mixture (25 μL) consisted of 0.8 μL template, 1× mixture for real-time PCR, and 1 μL of each primer.

The quantitative assessment of gene expression was performed by the 2^−ΔΔCt^ method relative to the expression of the reference GAPDH gene. Real-time RT-PCR was performed in triplicate.

Primers are listed in Table 1 [21].

### 2.6. P-glycoprotein ATPase Assay

The Complete Set of Reagents for Performing Luminescent P-glycoprotein ATPase Assays with Pgp-Glo™ Assay System cat. #V3601 (Promega Corporation, Madison, WI, USA) was used to assess the effect of the compound on the ATP Binding Cassette Subfamily B Member 1 (MDR1, P-glycoprotein, EC 7.6.2.2) ATPase activity. The analysis was carried out in accordance with the manufacturer’s instructions; tariquidar instead of verapamil was used as a stimulator of the ATPase activity. Solutions of the studied substances were prepared in an Assay Buffer with 0.5% DMSO; Na_3_VO_4_ was used as an inhibitor of the P-glycoprotein ATPase activity; an untreated control was used to determine the basal activity.

The samples of the recombinant human P-glycoprotein membranes were incubated with MgATP in the presence of the compounds. During the 40 min incubation at 37 °C, some ATP was consumed by P-glycoprotein. A luciferase-based ATP Detection Reagent was then added to stop the P-glycoprotein activity and initiate a luciferase reaction that produces glow-type luminescence. The luminescence was measured after 20 min, using CLARIOstar^®^ microplate reader (BMG LABTECH, Inc., Cary, NC, USA).

### 2.7. Statistical Analysis

All experiments were conducted at least in triplicate. Statistical analysis was performed using Microsoft Excel 2010 and Origin 2019b. The results are presented as the mean ± SEM.

Statistical significance was assessed using a one-sample *t* test (Origin 2019b). All data are presented as the mean ± SEM from at least three independent replicates. *p* values < 0.05 were considered to indicate statistically significant results. Figures represent summaries of at least three independent experiments in proportion to the related control, if not otherwise specified. Microscopy data are shown as one representative out of three independent experiments.

## 3. Results and Discussion

### 3.1. Tumor Cells Adapt to Nutlin-3a Significantly Worse Than to Paclitaxel

At the present stage of antitumor therapy development, an important parameter is the ability of the drug to suppress the development of a small group of chemo-resistant cells that survive the course of therapy and initiate the development of a secondary tumor or metastases. In our efforts to establish stable cell lines suitable for such in vitro testing, we worked with chemo-resistant cells based on the human colon cancer cell line HCT116, which is widely used in therapeutic research and drug screening, and noted significant differences in cell adaptation to drugs with different mechanisms of action [21]. Over a year, we increased the concentrations of the drugs in the medium, pursuing cell adaptation to paclitaxel and Nutlin-3a. During this time, we managed to increase the concentration of paclitaxel in the nutrient medium by 200 times (from 0.01 µM to 2 µM), while at the same time, under similar conditions, we failed to obtain a stable HCT116 strain resistant to 60 µM of Nutlin-3a (against the initial concentration of 10 µM); the cells died after several passages under these conditions.

Such data allow us to expect a lower risk of developing chemo-resistance during therapy with a targeted agent, when compared with a cytostatic agent. To study the mechanisms underlying such significant differences, we compared the obtained cell lines using the following strains as an example:

- HCT116_nut_—HCT116 resistant to Nutlin-3a at a concentration of 30 μM;
- HCT116_tax_—HCT116 resistant to paclitaxel at a concentration of 0.1 μM.

**Figure 1:**
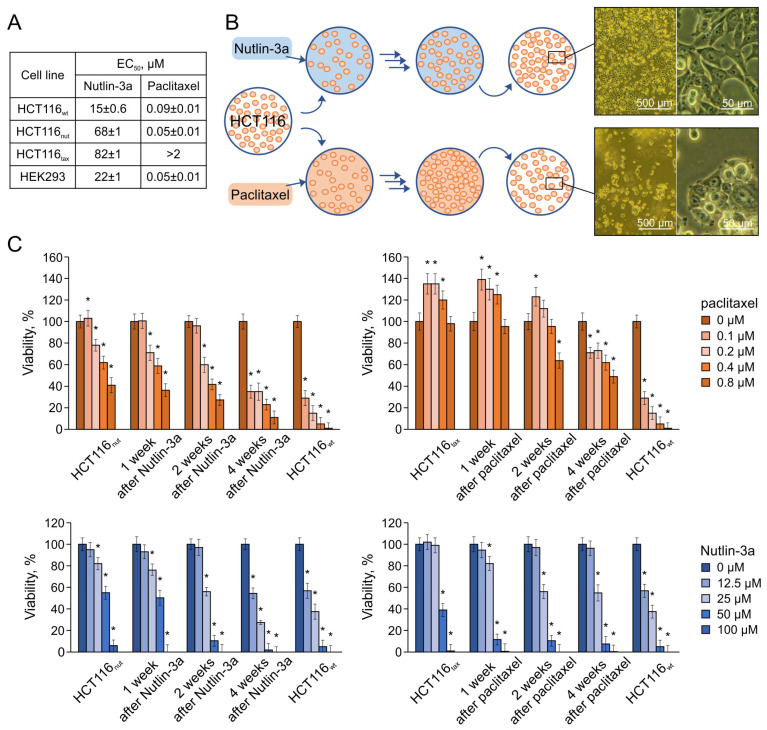
The sensitivity of cell lines to paclitaxel and Nutlin-3a. (**A**) The EC_50_ of drugs according to the MTT test. (**B**) The scheme of cell cultivation in the presence of drugs and after their removal from the medium. The pictures show HCT116_nut_ (**top**) and HCT116_tax_ (**bottom**) 2 days after inoculation into the drug-free medium; cells selected on Nutlin-3a adhered well and began to grow actively; cells after paclitaxel showed signs of stress. (**C**) Changes in cell susceptibility to drugs after 1, 2, and 4 weeks of cultivation in a normal nutrient medium. Survival in the absence of drugs on the corresponding day was taken as 100%. Data shown are the mean of 3 independent experiments ± SEM. * *p* < 0.05 vs. corresponding control; Student’s *t* test.

These strains grow stably in the presence of ≈5 × GI_50_ of the corresponding drug [21], which allows us to consider them conditionally equivalent. Such drug concentrations are toxic both for the maternal HCT116_wt_ line and for the cell line derived from human embryonic kidney cells HEK293 (Figure 1A); paclitaxel, unlike Nutlin-3a, suppresses the latter even more effectively than classical tumor cells.

As mentioned above, cells that have undergone adaptation are able to withstand several passages in the presence of 60 μM Nutlin-3a, but will gradually die, which excludes the possibility of their long-term study. However, the considered concentrations of drugs (30 μM Nutlin-3a and 0.1 μM paclitaxel) allow for the stable cultivation of cells after an adaptation period.

We examined the response of HCT116_nut_ and HCT116_tax_ cells to drug withdrawal. For this purpose, cells were plated at a concentration of 1,200,000 cells/mL on dishes with DMEM supplemented with *L*-glutamine and FBS without the drugs. After just two days, the following significant difference in the behavior of the cultures was noted: while the cells selected on Nutlin-3a adhered well and began to grow actively, the cells after paclitaxel, on the contrary, clearly showed signs of stress in the absence of the selection drug (Figure 1B).

To study the loss of the resistance of the resulting strains, their survival was quantified when treated with drugs after one, two, and four weeks [28]. The data obtained demonstrate that cells lose resistance to Nutlin-3a within a month (Figure 1C). At the same time, cells that have adapted to paclitaxel not only maintain resistance for a long time, but their growth is, on the contrary, stimulated in the presence of the drug.

Thus, cells selected by drugs with different mechanisms of action behave in fundamentally different ways. The drugs not only demonstrate a fundamentally different range of potential resistance, but also provoke tolerance of varying durations.

### 3.2. Nutlin-3a Sensitizes Cells to Drugs of Other Mechanisms, While Paclitaxel Provokes Multidrug Resistance

The most dangerous manifestation of resistance is the development of multidrug resistance, when the tumor becomes resistant to a wide range of drugs with different mechanisms of action. Currently, multidrug resistance is one of the main problems in the treatment of patients with various cancers [29]. To identify such consequences, we examined the sensitivity of the resulting sublines to Nutlin-3a, paclitaxel, as well as to an Mdm2 inhibitor based on isoindolinone I-I [12,30] and to the proteasome inhibitor MG132 [31,32]. To avoid combined effects, one day before the experiment, the medium with the corresponding drug was replaced with DMEM, supplemented with *L*-glutamine and 10% FBS.

**Figure 2:**
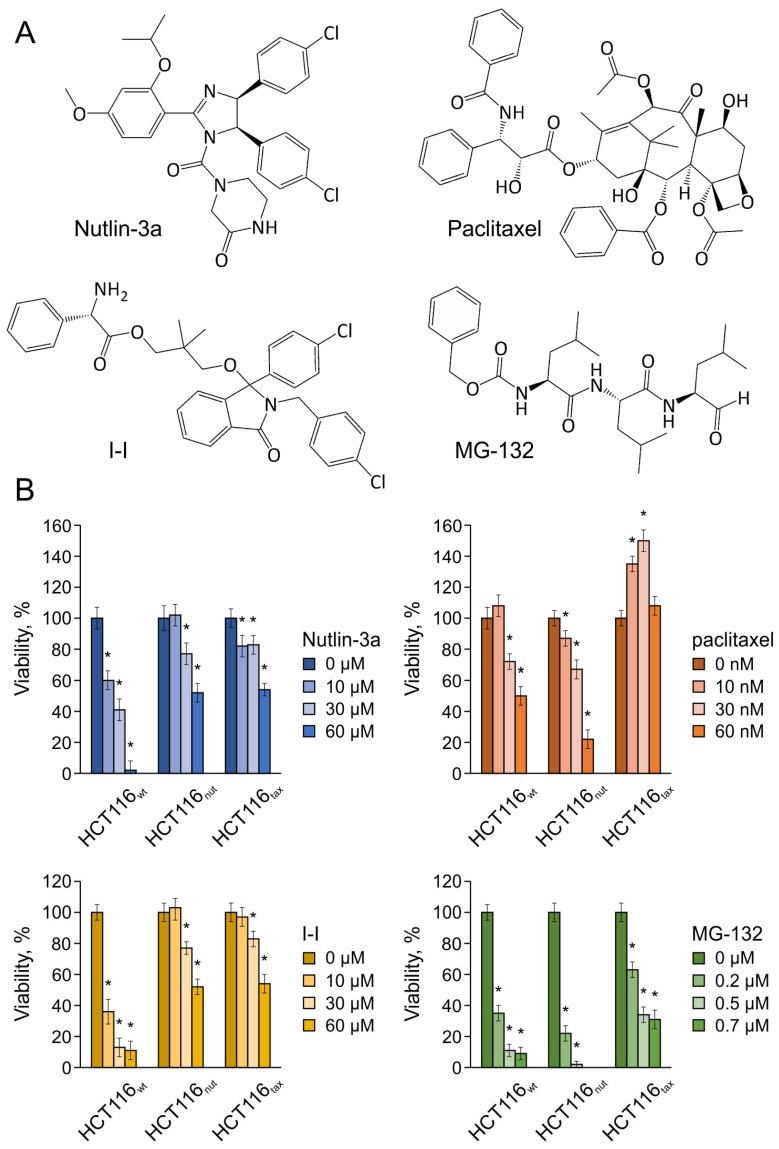
The effect of various classes of antitumor agents on the growth of HCT116_wt_, HCT116_nut,_ and HCT116_tax_ cells: (**A**) the structures of considered compounds; (**B**) the survival of different strains when treated with p53-Mdm2 inhibitors Nutlin-3a and I-I, chemotherapy drug paclitaxel, and proteasome inhibitor MG132. Data shown are the mean of 3 independent experiments ± SEM. * *p* < 0.05 vs. corresponding control; Student’s *t* test.

The data obtained using two resistant lines and the maternal line are presented in Figure 2. In the case of the Mdm2 inhibitors, both cell lines (HCT116_nut_ and HCT116_tax_) showed a similar sensitivity profile; however, when using drugs with other mechanisms of action, diametrically opposite effects were observed. The developed resistance to paclitaxel correlated with resistance to all drugs considered, regardless of their mechanism of antitumor action, which indicates the development of a multidrug resistance phenotype and is consistent with the literature data [33,34]. Moreover, we first showed that cells not only acquire resistance to the drug in the case of paclitaxel, but the presence of the drug in the environment stimulates cell growth rather than destroying them. In the case of the resistant line, a decrease in survival was observed only when the drug concentration was increased to 100 nM.

Long-term cultivation in the presence of Nutlin-3a naturally provoked the development of the resistance of HCT116_nut_ cells to Mdm2 inhibitors of various chemical structures, which is consistent with the data of [11], whereby a cross-resistance to MI-63 and Nutlin-3a was demonstrated. However, this adaptation made HCT116_nut_ cells significantly more susceptible to both paclitaxel and to the proteasome inhibitor MG132. The data obtained indicate not only a lower risk of developing resistance when using targeted drugs, but also allow us to expect good results from combined therapy based on the targeted drugs, as well as allowing us to rely on a positive outcome with the sequential use of various agents following Mdm2 inhibitors.

### 3.3. The Relationship between Adaptation to Nutlin-3a and ABC Transporter-Mediated Efflux Typical for Paclitaxel

Cells can develop various mechanisms of adaptation to chemotherapy, but the most predominant and universal resistance mechanism is the efflux of drugs by membrane transporters, such as P-glycoprotein and other members of the ATP-binding cassette (ABC) transporters [35,36]. The upregulation and the gain-of-function of ABC transporters is one of the important factors leading to MDR [37].

**Figure 3:**
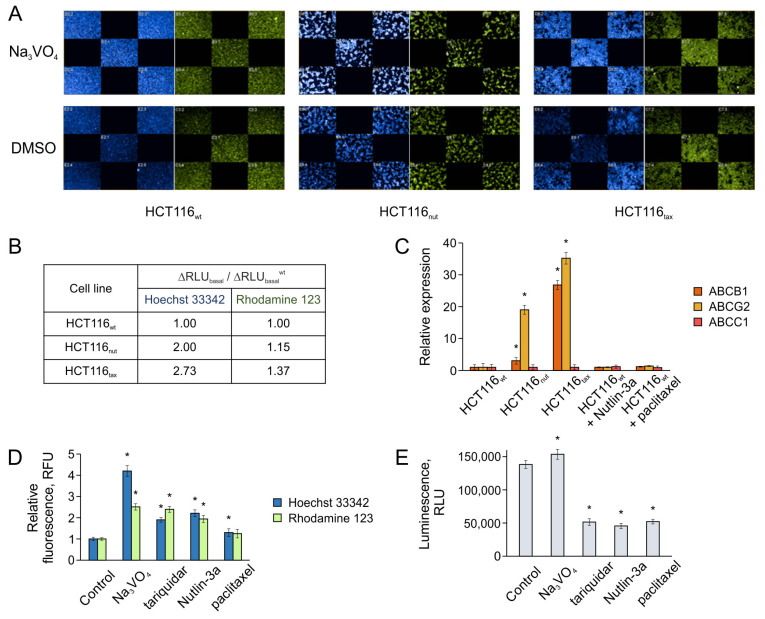
The role of ABC transporters in HCT116_wt_, HCT116_nut_, and HCT116_tax_ cells. Changes in cell fluorescence upon inhibiting ABC transporters using sodium orthovanadate: (**A**) snapshots (Operetta CLS™ High Content Analysis System) and (**B**) basal fluorescence levels relative to wild-type cells (CLARIOstar microplate reader). Hoechst 33342 (blue) and Rhodamine 123 (green) fluorescence was analyzed. (**C**) The relative gene expression of transport protein genes *ABCB1* (MDR1), *ABCG2* (BCRP), and *ABCC1* (MRP1) in HCT116_wt_, chemoresistant phenotype lines, and HCT116_wt_ treated with Nutlin-3a (5 μM) and paclitaxel (50 nM) for 48 h. The effect of compounds on (**D**) the accumulation of fluorescent substrates in HCT116 cells resistant to 1 mM paclitaxel and (**E**) the ATPase activity of MDR1. The following concentrations were used: 500 μM Na_3_VO_4_, 5 μM tariquidar, 1 μM paclitaxel, and 5 μM Nutlin-3a. Data shown are the mean of 3 independent experiments ± SEM. * *p* < 0.05 vs. corresponding control; Student’s *t* test.

In this work, to study the changes in the transport activity associated with the development of resistance, we assessed the basal transport activity of HCT116_wt_, HCT116_nut,_ and HCT116_tax_ cells. When studying the transport activity, the ability of cells to accumulate/efflux various ABC transporter substrates was analyzed over a fixed time. We considered two fluorescent substrates of the most common transporters, Hoechst 33342 (blue) and Rhodamine 123 (green) [21,38], at concentrations of 1 and 1.25 μM, respectively (Figure 3A). The comparison of cell luminescence under normal conditions and when treated with sodium orthovanadate, which completely suppresses the activity of efflux transporters, allowed us to estimate the basal level of efflux for the cells (∆RLU_basal_).

The data obtained confirmed that in HCT116_tax_ cells resistant to paclitaxel, the transport activity is significantly increased; sodium orthovanadate under such conditions causes a more significant change in the cell luminescence when compared with wild-type cells, which indicates an active constant release of substrates (Figure 3A). In the case of cells growing in the presence of Nutlin-3a, there was a similar but less pronounced effect.

Colorectal tumors are characterized by the increased gene expression of ABC transporters, such as MDR1 (*ABCB1*), BCRP (*ABCG2*), and MRP1 (*ABCC1*) [39,40,41,42]. In particular, these transporters release drugs such as doxorubicin, paclitaxel, etc. [43,44,45]. We also previously noted that an increase in the expression level of *ABCB1* is observed in HCT116 cells in response to long-term treatment with Nutlin-3a [21]. In this work, we used real-time RT-PCR to assess the expression levels of all three transporters (Figure 3C).

Although, according to the literature, MRP1 can cause chemoresistance in colorectal cancer cells [42], the expression of its *ABCC1* gene is not activated in the HCT116 line by either paclitaxel or Nutlin-3a. However, an increase in *ABCG2* expression (BCRP) was observed in both resistant strains.

The findings suggest that paclitaxel insensitivity is associated with the overexpression of both *ABCB1* (MDR1) and *ABCG2* (BCRP) transporters. This result, on the one hand, is consistent with the literature data [46,47], and on the other, it emphasizes the urgent need to expand the range of efflux proteins studied, compared with the recent idea of P-glycoprotein as the main source of resistance.

*ABCG2* (BCRP) is not only dominant in HCT116_tax_ cells, but is also almost solely activated in HCT116_nut_, although expressed 1.5 times less strongly than in HCT116_tax_. The level of *ABCB1* (MDR1) increases slightly as a result of the growth on Nutlin-3a; this is six times lower than *ABCG2* (Figure 3B). It is worth noting that the difference in transporter expression correlates well with the results of the fluorescent substrate accumulation assay (Figure 3A), since Rhodamine is thought to be transported primarily by MDR1, while the release of Hoechst 33342 is associated mainly with BCRP [48,49].

In addition to the effects of long-term cultivation, we also assessed the effects of short-term drug treatment on transporter gene expression and transport activity. The treatment of wild-type HCT116 cells did not increase transporter expression over either 24 or 48 h (Figure 3C). However, this time was sufficient for changes in cell transport activity under the influence of paclitaxel and Nutlin-3a (Figure 3D).

The activity of ABC-mediated efflux can be directly regulated by the interaction of transport proteins with specific drugs, with some substances (sodium orthovanadate, third-generation MDR1 inhibitor tariquidar) suppressing efflux, and others (ligands released by transporters) activating it. We compared the effects of paclitaxel and Nutlin-3a on the transport activity of HCT116 cell line resistant to 1 mM paclitaxel, using sodium orthovanadate and tariquidar as controls [13]. This line demonstrates significantly higher expression levels of both *ABCB1* and *ABCG2*, and is most responsive to the use of transport activity modulators, making it a convenient model for such tests.

Paclitaxel had a moderate effect on the transport activity of cells as follows: even when using 1 μM of the drug, i.e., at 100-fold excess of the IC_50_ value, no significant accumulation of fluorescent substrates was observed. At the same time, Nutlin-3a, at a concentration corresponding to its IC_50_ (5 μM), significantly suppressed the transport activity, as evidenced by the accumulation of the fluorescent substrate in cells comparable to the effect of tariquidar (Figure 3D). This result is consistent with the data on the applicability of Mdm2 inhibitors for the development of MDR1 inhibitors [15], and confirms that such agents may be promising for the combined therapy.

The efflux process is associated with the hydrolysis of ATP molecules, which is necessary for the implementation of conformational rearrangements of the transporter, determining the transfer of substances across the cell membrane. In this case, the binding of substances to the transporter can either stimulate or suppress its ATPase activity. 

We used the Pgp-Glo™ assay system to study the effect of Nutlin-3a and paclitaxel on the ATPase activity of MDR1. Sodium orthovanadate was again used as a control, since it completely inhibits the ATPase activity of MDR1 [50]. Other inhibitors of MDR1-mediated efflux, such as tariquidar and verapamil, on the contrary, activate the ATPase activity of the transporter, but at the same time, suppress the efflux of other substrates (such as Rhodamine 123), interfering with the latter [51,52].

Nutlin-3a and paclitaxel, in contrast to sodium orthovanadate, stimulate the ATPase activity of the transporter (Figure 3E). Since both substances suppress the efflux of Rhodamine 123 and Hoechst 33342 (Nutlin-3a is significantly more effective, Figure 3D), it can be argued that there is a direct binding of the agent to the transporter, which leads to the suppression of the efflux of other substances. Thus, Nutlin-3a and paclitaxel act similarly to tariquidar, with the effect of paclitaxel being negligible, while Nutlin-3a has an effect comparable to the classical MDR1 inhibitor.

Thus, the use of Nutlin-3a not only does not lead to the activation of the expression of transporter genes, which is the key cause for the development of MDR, but also suppresses their ability to efflux other substances, such as fluorescent substrates or anticancer drugs.

### 3.4. Nutlin-3a Provokes Changes in p53-Mediated Processes and Promotes the Apoptosis of Tumor Cells during Combined Therapy

**Figure 4:**
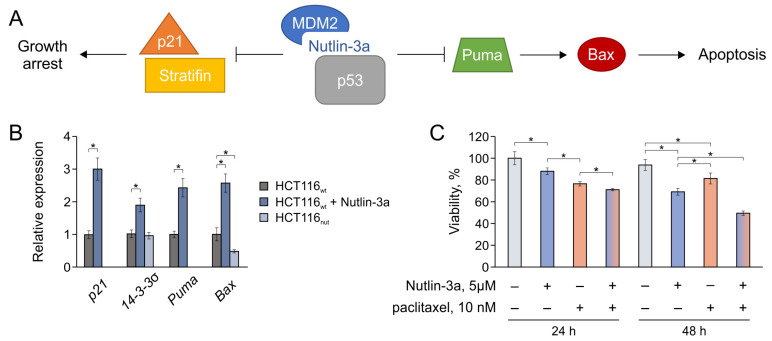
The effect of Nutlin-3a on HCT116 cells. (**A**) Pathways for Mdm2 inhibitor Nutlin-3a affecting the cell: *p21*, *Puma*, *Bax*, *14-3-3σ* (Stratifin). (**B**) Relative gene expression in HCT116 cells when cultured with Nutlin-3a for 24 h and 12 months (HCT116_nut_). (**C**) The individual and combined effects of Nutlin-3a and paclitaxel on HCT116_wt_ cells. Data shown are the mean of 3 independent experiments ± SEM. * *p* < 0.05; Student’s *t* test.

As noted above, in the case of Mdm2 inhibitors, resistance is usually associated with changes in p53-mediated processes, which naturally stems from the mechanism of the action of such compounds. Therefore, we studied the effect of Nutlin-3a on the transcription of key p53 target genes, including *p21*, *Puma*, *Bax*, and *14-3-3σ* (Stratifin) (Figure 4).

P21, cyclin-dependent kinase inhibitor 1A, is a well-known p53 target gene, which plays a critical role in the regulation of p53-dependent cellular senescence by inducing G1/S-phase cell cycle arrest [53,54]. Puma, a p53 upregulated modulator of apoptosis (PUMA), a pro-apoptotic BCL-2 homology 3 (BH3)-only member of the BCL-2 family, is a direct transcriptional target of p53 that induces mitochondrial apoptosis [55]. Bax is a BCL-2 family protein that forms a heterodimer with BCL-2, and functions as an apoptotic activator [56]. Stratifin, the σ isoform of 14-3-3, inhibits G_2_/M cell cycle progression in a p53-regulated manner, and is critical to uphold G_2_ arrest upon DNA damage in colorectal cancer cells [57].

We compared the effects in the case of the short-term treatment of HCT116_wt_ cells with the drug and long-term cultivation (HCT116_nut_). The treatment with Nutlin-3a for 24 h (5 µM) expectedly leads to an increase in the transcription levels of all genes considered by the reactivation of p53, due to the disruption of the p53-Mdm2 interaction (Figure 4B). In turn, in the resistant HCT116_nut_ line, the expression of the considered genes is reduced, which prevents the cascade of processes that should lead to cell cycle arrest and apoptosis. The observed gene expression profile, in which *p21* and *Puma* transcripts are not detected and *Bax* is reduced by 2 times, indicates the presence of disturbances in the early stages of p53-mediated processes, which is common for the considered targets.

The observed pattern explains the limited adaptation of cells to Nutlin-3a, compared with the response to paclitaxel. While the expression of efflux transporters in cells in response to the cytostatic drug paclitaxel can increase 20-fold or more (Figure 3C), the expression of p53 target genes can decrease only in a small range (Figure 4B), and this threshold is apparently reached at Nutlin-3a concentrations > 30 µM, when cells slowly but surely die.

The presented data suggest that the use of Mdm2 inhibitors should not only be safer from the point of view of the development of chemoresistance, but should also help to overcome paclitaxel-mediated chemoresistance via the suppression of ABC transporter-mediated efflux. To evaluate the combined effect of drugs, we analyzed the survival of HCT116_wt_ in the presence of individual drugs and their mixture (Figure 4C). During the first 24 h, paclitaxel exhibits its cytotoxic effect, while Nutlin-3a does not have time to significantly implement the mechanisms of p53-mediated cell death. The combined effect observed in this case should be associated with the suppression of ABC-mediated paclitaxel efflux by the action of Nutlin-3a as a transporter inhibitor.

On the second day, the situation changes significantly. During this time, the solo effect of Nutlin-3a has time to develop; however, in the case of paclitaxel, the survived cells begin to successfully adapt (no subsequent cell loss is observed). The combined use of drugs within 48 h gives a noticeable effect, preventing adaptation to paclitaxel and allowing for the implementation of several antiproliferation mechanisms at once. Thus, the combined use of paclitaxel and Nutlin-3a reduces the risks of tumor cell adaptation and, consequently, the risks of developing secondary chemoresistance.

The whole data obtained allow us to get an idea of the differences in the development of chemoresistance in the case of administering targeted therapy and cytostatics. While the tolerance to paclitaxel is associated with the development of multidrug resistance, which has a negative prognosis for patients, in the case of Nutlin-3a, cells not only rapidly lose their drug resistance properties when the treatment is stopped, but also become more susceptible to agents with a different mechanism. The observed effects are associated both with the different profiles of launched cell defense mechanisms, and with the ability of Nutlin-3a to act as an efflux inhibitor.

## 4. Conclusions

This work is the first to examine the cultivation of cells in the presence of the targeted antitumor agent Nutlin-3a for months. Our data clearly indicate that targeted therapy with p53 reactivators is much safer in terms of the development of secondary chemoresistance than the use of the cytostatic drug paclitaxel. Understanding the processes occurring in cells during long-term treatment will allow for the rationalization of the choice of regimens and for the development of new agents.

## Figures and Tables

**Table 1:** Primer sequences for the studied genes.

Gene	Protein	Forward	Reverse
*ABCB1*	MDR1	GGGAGCTTAACACCCGACTTA	GCCAAAATCACAAGGGTTAGCTT
*ABCC1*	MRP1	TCAGGAGCACACGAAAGTCC	AAGAAGCTCATGGGTGACCG
*ABCG2*	BCRP	AAGCCACAGAGATCATAGAGCC	TCTTCTTCTCACCCCCGGAA
*P21*	P21	ACTGTCTTGTACCCTTGTGCC	AAATCTGTCATGCTGGTCTGC
*Puma*	Puma	GCGAGACTGTGGCCTTGTGT	CGTTCCAGGGTCCACAAAGT
*Noxa*	Noxa	GCTGGAAGTCGAGTGTGCTA	TTCCTGAGCAGAAGAGTTTGG
*Bax*	Bax	CCGAGAGGTCTTTTTCCGAG	CCAGCCCATGATGGTTCTGAT
*14-3-3σ*	Stratifin	CATCATTGACTCAGCCCGGT	AGTGTCAGGTTGTCTCGCAG
*GAPDH*	GAPDH	GGGAAGGTGAAGGTCGGAGT	TTGAGGTCAATGAAGGGGTCA

## Data Availability

Data are contained within the article.
